# Characterization of essential oil distribution in the root cross-section of *Valeriana officinalis* L. s.l. by using histological imaging techniques

**DOI:** 10.1186/s13007-018-0309-4

**Published:** 2018-06-04

**Authors:** Michael Penzkofer, Andrea Baron, Annette Naumann, Andrea Krähmer, Hartwig Schulz, Heidi Heuberger

**Affiliations:** 1 Institute for Crop Science and Plant Breeding, Research Group Medicinal and Spice Plants, Bavarian State Research Center for Agriculture (LfL), Am Gereuth 2, 85354 Freising, Germany; 2 Federal Research Centre for Cultivated Plants, Institute for Ecological Chemistry, Plant Analysis and Stored Product Protection, Julius Kühn-Institute (JKI), Königin-Luise-Straße 19, 14195 Berlin, Germany

**Keywords:** Valerian, Medicinal plant, Root slice, Thin-section, Oil droplet, Fluorescence-microscopy, Fourier-transform infrared (FTIR) spectroscopy, Nile Blue A, Sudan-III

## Abstract

**Background:**

The essential oil is an important compound of the root and rhizome of medicinally used valerian (*Valeriana officinalis* L. s.l.), with a stated minimum content in the European pharmacopoeia. The essential oil is located in droplets, of which the position and distribution in the total root cross-section of different valerian varieties, root thicknesses and root horizons are determined in this study using an adapted fluorescence-microscopy and automatic imaging analysis method. The study was initiated by the following facts:

- A probable negative correlation between essential oil content and root thickness in selected single plants (elites), observed during the breeding of coarsely rooted valerian with high oil content.
- Higher essential oil content after careful hand-harvest and processing of the roots.

**Results:**

In preliminary tests, the existence of oil containing droplets in the outer and inner regions of the valerian roots was confirmed by histological techniques and light-microscopy, as well as Fourier-transform infrared spectroscopy. Based on this, fluorescence-microscopy followed by image analysis of entire root cross-sections, showed that a large number of oil droplets (on average 43% of total oil droplets) are located close to the root surface. The remaining oil droplets are located in the inner regions (parenchyma) and showed varying density gradients from the inner to the outer regions depending on genotype, root thickness and harvesting depth.

**Conclusions:**

Fluorescence-microscopy is suitable to evaluate prevalence and distribution of essential oil droplets of valerian in entire root cross-sections. The oil droplet density gradient varies among genotypes. Genotypes with a linear rather than an exponential increase of oil droplet density from the inner to the outer parenchyma can be chosen for better stability during post-harvest processing. The negative correlation of essential oil content and root thickness as observed in our breeding material can be counteracted through a selection towards generally high oil droplet density levels, and large oil droplet sizes independent of root thickness.

**Electronic supplementary material:**

The online version of this article (10.1186/s13007-018-0309-4) contains supplementary material, which is available to authorized users.

## Background

Valerian (*Valeriana officinalis* L. s.l.) is an herbaceous perennial plant with a huge variability regarding habitus, composition of ingredients, and agro-economic traits. The leaves usually are imparipinnate, and the leaflets, weakly to strongly serrated. For blooming, a vernalization is necessary and hence, the first inflorescence usually develops in the second year of cultivation. Valerian occurs on sporadically wet habitats in the temperate zone of the northern hemisphere. This indicates that a secure water supply is necessary for cultivation. Usually, the rootstock forms a dense meshwork of thin roots [1].

For medicinal purposes, the entire root system including the rhizome is used [2]. Preparations based on valerian roots are used against restlessness and sleep disturbances [3]. In Germany, the dried root of valerian is a component of about 86 phytopharmaceutical and homeopathic preparations. In North America (USA, Canada, Mexico), due to other admission procedures, more than 1000 products with valerian root are obtainable. In Germany alone, the demand for dried roots amounts to app. 1000 tons, equal to a market size of app. 4 Mio.€ [4–8].

To counteract the losses of root mass and secondary compounds during harvesting, cleaning and the further production process of dried valerian roots, breeding was started in 2008 to develop new varieties of valerian with a coarser root-system (thicker adventitious roots) and with high contents of secondary compounds. A coarser root system would probably preserve the secondary compounds, essential oil and valerenic acid [9]. According to the European Pharmacopoeia, the minimum content of essential oil must be 4 ml  kg^−1^ and of valerenic acid at 0.17% (m/m) [2]. The most frequent major constituents of essential oil of *Valeriana officinalis* L. s.l. are the monoterpenes borneol and its esters, bornyl acetate and bornyl isovalerate [10–15].

In contrast to the abundant analyses of pharmaceutical secondary compounds and their medicinal values, there are relatively few studies related to the physiology and localization within the root. Zacharias [16] described essential oil to be located in ‘‘[…] the outer exodermis […]’’, whereas Tschirch and Oesterle [17] found it in the ‘‘[…] single-row hypodermis […]’’. Both authors described one ‘oil droplet’ in a single exodermis cell. Localization of essential oil only in the outer cell layers of the valerian roots would support the two following observations made during the breeding of coarse valerian: (i) Considering 200 selected plants (elites), the essential oil content decreased with the increase of root thickness [18]. This behavior is explainable, because with increasing root diameter, the root surface area decreases in relation to the root volume (calculated as cylinder). (ii) After careful hand-harvesting and hand-processing, high essential oil contents were achieved [19]. Due to careful handling, the surface was not damaged and the essential oil, close to the root surface, still present. The presence of essential oil close to the root surface was confirmed by Holzner-Lendbrandl [20] und Fridvalszky [21], who additionally recognized small round bodies named ‘oil sacs’ in the parenchyma of the roots. These ‘oil sacs’ were found predominantly in the outer parenchyma. Violon et al. [22] identified ‘oil droplets’ also in the inner parenchyma. All previous investigations remain vague about the oil droplet identification and distribution across the cross-section. In addition, they do not give information concerning oil droplets among different varieties, at different root diameters on the same plant, or at different positions along the roots.

The application of various vibrational spectroscopy methods for visualizing secondary metabolites in different plant tissue is already described for e.g. polyacetylenes and carotenoids in carrots, or essential oil components in fennel, chamomile and curcuma [23–28]. The Fourier-transform infrared FITR imaging method allows one to study the occurrence and distribution of a wide range of molecules in cell tissues. However, it has not yet been applied for the essential oil in valerian. The fluorescence-microscopic method is suitable for the visualization and localization of secondary compounds in plant roots, and was used with sunflowers and mountain arnica [29–31]. Furthermore, a spectral-sensitive camera could make more oil droplet structures visible, or make chemical differentiation possible, respectively [32, 33].

Our intention was to give a more detailed histochemical description within the valerian roots. The development of an appropriate method to visualize and clearly identify the essential oil droplets required several consecutive steps grouped into two fields: (k) Verification of oil droplets and (kk) generation of an essential oil distribution map. Verification of the oil droplets (k) was done by light-microscopic imaging and subsequent confirmation of the essential oil in the found oil droplets by Fourier-transform infrared (FTIR) imaging. Based on the results of these investigations, fluorescence-microscopic imaging for generating oil droplet maps (kk) could be applied.

Based on this, the study at hand was carried out to better understand the histochemical background of the two observations (i) and (ii). Observation (i) must be well interpreted to assess the achievability of the breeding target of a thick root-system with good essential oil content. Understanding the histological background of observation (ii) may explain why the entire essential oil is not all lost during a more robust, mechanized root harvesting and processing in large-scale valerian field production. We postulated that a great part of the essential oil droplets occur in the inner parts of the valerian root.

## Methods

### Plant material

In 2010, three elite plants were selected from the variety ‘Anton’ (seed source: N.L. Chrestensen Erfurter Samen- und Pflanzenzucht GmbH, Erfurt, Germany, 2008) and one elite plant from the variety ‘Lubelski’ (seed source: PHARMASAAT Arznei- und Gewürzpflanzensaatzucht GmbH, Artern, Germany, 2009) based on their differing root morphology and essential oil contents (Table 1). Two elites were characterized as thin-rooted, meaning that they predominantly formed a highly branched and felted rootstock with thin adventitious roots. The other two elites predominantly formed a chunkier and less felted rootstock with thicker adventitious roots; these were called thick-rooted.

**Table 1:** Root morphology types and content of essential oil in the root drug of the cloned elite plants (CE)

Cloned elites	Varieties	Morphological root structure	Essential oil^a^	Root fraction (diameter)			
		Classification	ml kg^−1^	RF1 (< 2 mm)^b^	RF2 (< 3 mm)^b^	RF3 (< 4 mm)^b^	RF4 (> 4 mm)^b^
CE1	‘Anton’	Thin-rooted	8.9 (14.1)	●	●	●	—
CE2	‘Anton’	Thin-rooted	10.4 (19.9)	●	●	●	
CE3	‘Anton’	Thick-rooted	5.0 (12.9)	●	●	●	●
CE4	‘Lubelski’	Thick-rooted	6.9 (11.3)	●	●	●	●

Prevalence of root thickness fractions (RF) in the clone plants

^a^Essential oil content of the mother plant (elites) and in brackets the cloned elites, determined in 2010 and 2012, respectively

**Fig. 1:**
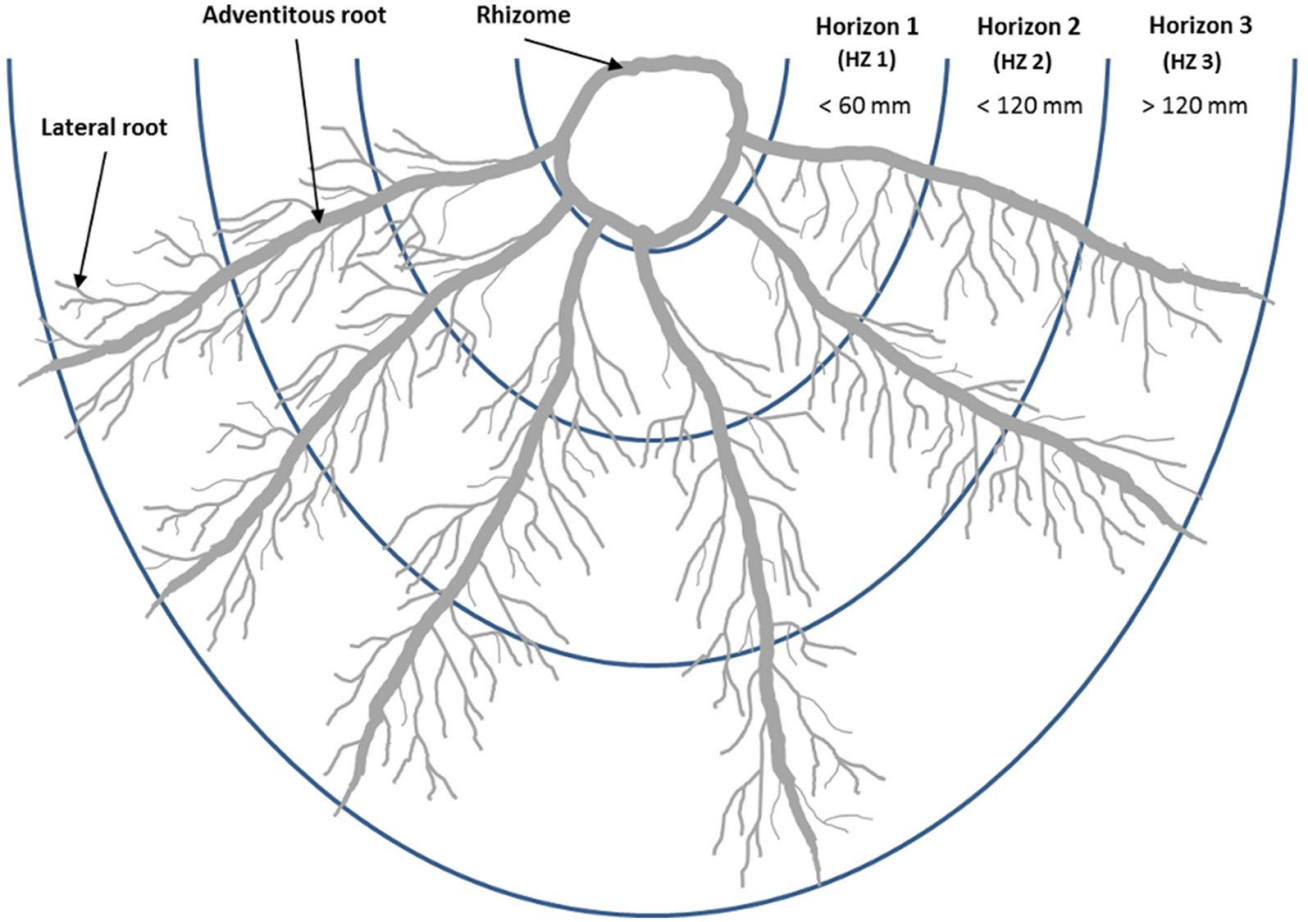
The valerian root system components and visualization of the investigated horizons

^b^Diameter at the base (Horizon 1, see Fig. 1) is decisive for the classification to a fraction. The ● indicates the formed and the — indicates the not formed fractions

In contrast to common cultivation, in the current study, clones were used in order to have plants with known analytical and identical genetic backgrounds. Plants of seed propagated valerian populations would probably vary too much in the contents of secondary compounds [34]. Therefore, the four elite plants were cloned by sterile micropropagation using inflorescences at an early bud stage as starting tissue and side shoots as propagation parts. Rooted plantlets were cultivated for 2 years in the field of the experimental station Baumannshof of the Bavarian State Research Center for Agriculture (48°42′N /11°32′E, 360 m MSL). A detailed description of the plant material, the cloning by in vitro propagation and the cultivation conditions are described in Penzkofer et al. [19], where the same plant material was used.

Due to the age of the plants, flowering was induced and shoots started to develop in spring of the harvest year. The inflorescence development influences the essential oil content and causes a decrease of the essential oil content from summer to autumn [35]. Therefore, the inflorescences were cut off in an early stage of development to counteract the decline [36]. Our plant material was harvested in autumn (2014), the usual harvest time for valerian cultivation. At least one cloned plant of each elite was dug out carefully and the adventitious roots were separated into four diameter fractions (< 2, <  3, < 4, > 4 mm; Table 1). The fresh roots were stored in air-tight containers at 5–6  °C to prevent dehydration of the roots and a loss of essential oil. Prior to preparation for microscopy, the roots were washed carefully with water.

### Imaging methods

#### Verification of oil droplets (k)

##### Classic light-microscopic imaging

To confirm literature observations we used the classic method for microscopic imaging by fixing the cell components with a formaldehyde-propionic acid–ethanol-solution (5–5–90% FPA) and embedding the fixed roots in historesin (2-hydroxyethyl methacrylate). The starch was colored with a Lugol’s solution (potassium tri-iodide) and washed out with potassium hydroxide (KOH).

Thin slices of embedded roots, as well as thin longitudinal-section slices of root parenchyma and exodermis from fresh valerian roots, were colored with sudan-III-solution to make the lipids visible [37]. The sections were evaluated with a light microscope (ZEISS Axiostar plus, Carl Zeiss AG, Oberkochen, Germany) at magnification of 200.

##### Fourier-transform infrared (FTIR) imaging

Thin-section slices of 0.01 mm thickness were made by a freezing microtome (Leica CM 1100, Leica Biosystems Nussloch GmbH, Nussloch, Germany). Each slice was screened for colorless round bodies by light microscopy that was integrated in the FTIR spectrometer. Sections with round bodies were subsequently analyzed applying FTIR imaging. FTIR transmission spectra of the thin-section slices were recorded with the FTIR spectrometer Varian 4100 FTIR (Agilent, Waldbronn, Germany) combined with the IR-microscope Varian UMA 600 (Agilent, Waldbronn, Germany). The thin-section slices were placed on a ZnSe window and FTIR images were produced with a 32 × 32 focal plane array detector (FPA). The spectra were recorded over the wavelength range of 4000–850 cm^−1^ and 128 scans per spectrum were accumulated.

The absorption band at 998 cm^−1^ represents mainly cellulose, whereas the signal at 1737 cm^−1^ was assigned to C=O vibration of bornyl acetate. Both signals were used to display the distribution in pseudo-color images.

#### Fluorescence-microscopic imaging and mapping of differing root material (kk)

##### Sample preparation and producing of thin root-slices

From each cloned elite (CE) and root fraction (RF), two well-developed fresh roots with the typical root appearance were chosen and classified into three approximately 60 mm long parts, called horizons (HZ1 to HZ3, Fig. 1). HZ1 represents the rhizome-near part, which would certainly be harvested after cultivation, and HZ3 represents the rhizome-far part, which probably remains in the ground. Along the whole length of the horizons, several thin-sections were taken and prepared.

The cutting of thin root-slices was done by hand with a height adjustable cylinder-microtome. A segment of the respective root diameter fraction and horizon was clamped between a buffer-material, cut out from a carrot root parenchyma. This material has a comparable structure and consistency to the examined valerian roots. Thereby, the fresh valerian roots were well enclosed. With a moderate pressure, a constant velocity and without displacement, a straight metallic blade was moved through the valerian root tissue. The cut was performed in a constant angle of 20°. Through the moveable and height adjustable hanger, thin root-slices of a uniform thickness of 0.2 mm were able to be produced. These root-slices were then placed on top of a drop of water on a microscope slide. From each horizon, 15–20 thin-sections were cut, but just a low number of slices (seven on average) were suitable for the following image processing, analysis and evaluation.

##### Staining and fluorescence-microscopy

After the root-slices were cut, the water was removed and, based on the experiences of Fridvalszky [21], one to three drops of 1% aqueous solution of Nile Blue A (Carl Roth GmbH und Co. KG, Karlsruhe, Germany) were applied. After incubating the root-slices for one minute at room temperature, the solution was carefully removed. A drop of water was applied and the root-slices were covered with a cover glass. A further incubation period of at least 30 min followed.

The fluorescence-microscopy was done with a magnification of 100 (ocular 10×, objective 10×, ZEISS Axiostar plus, Carl Zeiss AG, Oberkochen, Germany). The initial light generated by a HBO50 high pressure mercury arc lamp was filtered for excitation at 430–510 nm and for emission at 475–575 nm. The images were taken with the connected digital camera ZEISS AxioCam ERc 5 s (a hyperspectral camera was not available) and instantly transferred to the connected computer. Due to the limited lens coverage, the final picture of a complete root-slice had to be composed of several partial images.

The green fluorescence oil droplets were imaged with a high-contrast against the black background. Despite all precautions taken, the root-slices did not always have exactly the same thickness and the cytoplasm of the cut cells was more or less leaked, so that the light transmittance of the cell layers varied among and within the root-slices. In order to make the oil droplets clearly visible in all areas of the root slice, brightness and contrast were adjusted through the camera software (ZEISS AxioVison, Carl Zeiss AG, Oberkochen, Germany) by one to two units, upwards or downwards, for each image. The depth of focus on the microscope was not changed so that the same cell layer was shown on each image.

##### Image processing

All partial images of one root slice were converted from the camera software’s own file format to the compressed free TIFF file format and then manually merged to one complete root-slice image with the image editing program Adobe Photoshop CS6 (Adobe Systems Software, Dublin, Republic of Ireland).

**Fig. 2:**
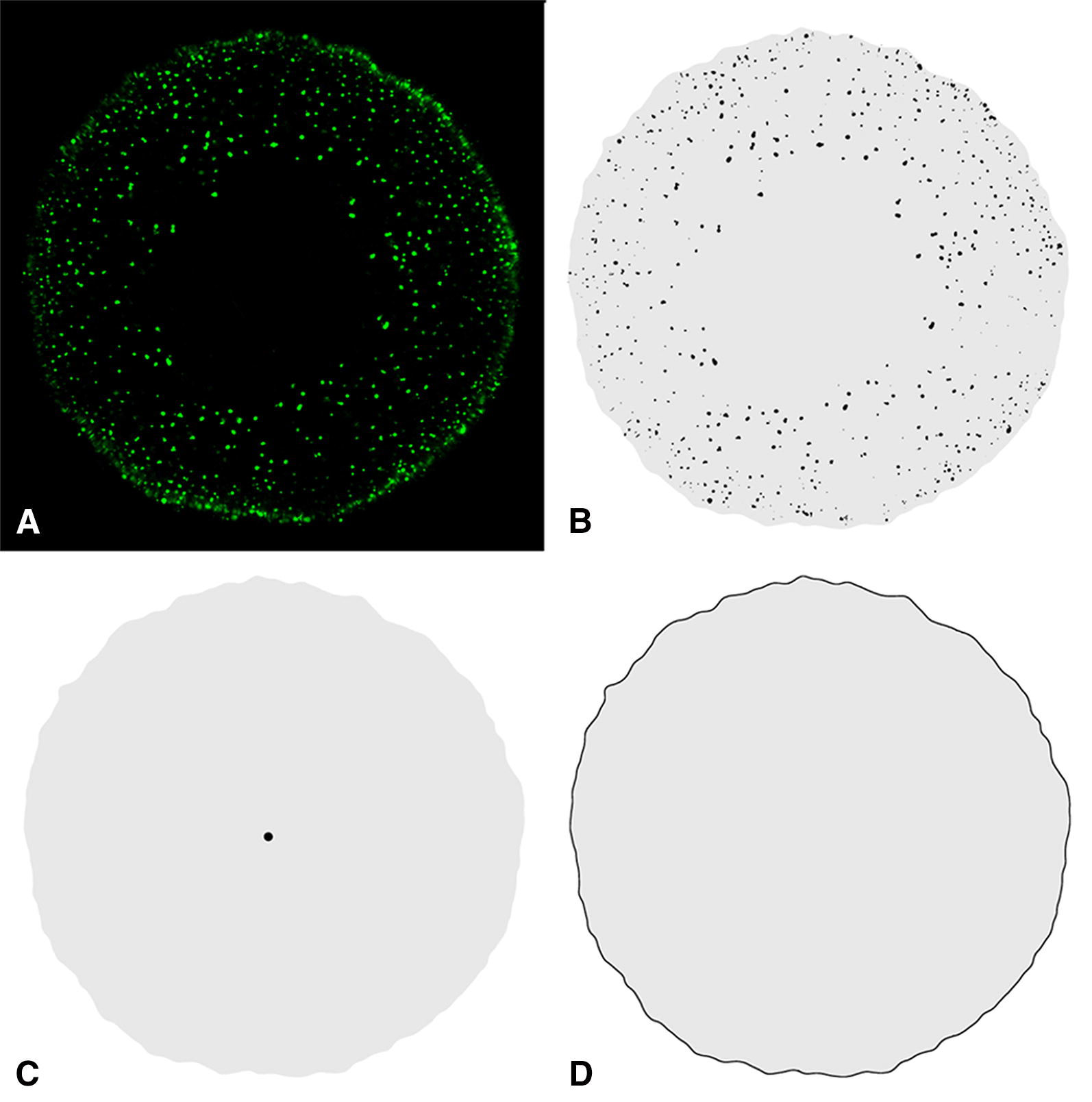
Different black-white-masks derived from the original composed root-slice image. **A** Root-slice image with green shining oil droplets. **B**–**D** Black-white masks of **B**: the oil droplets, **C** the center of the root slice, **D** the edge of the root-slice. The x–y-coordinates of the oil droplets and the center (black particles in **B** and **C**) and each point of the edge in **D**, and the size of the grey area (seen in **B**–**D**) were determined automatically by the image analysis software

The composed root-slice images were analyzed with the image analysis software ImageJ [38]. Software macros were developed to generate black-white-masks of the oil droplets, the root-slice center and the root-slice edge from each composed root-slice image (Fig. 2B–D). More information on the functionality is given in the Additional file 1.

The principle steps were to convert the composed image of the root slice into 8 bits grayscale image, to reduce the background noise using ImageJ image filtering operators (median, dilate), and segment the oil droplet by using the Huang Threshold method implemented in ImageJ.

Due to the variable position and the inconsistently round shape of the central cylinder, as well as the edge of the root-slice, the center of the root-slice was manually marked in the original composed root-slice image. After treating images that way, they were converted into a binary image (black and white) and x–y-coordinates of the now black particles were determined by using filters for particle size and particle circularity. The determination of the x–y-coordinates of the centers of the root slices and each point of the edges were done in a similar manner. Each image was treated with the same adjustments.

##### Data evaluation and statistical analysis

**Fig. 3:**
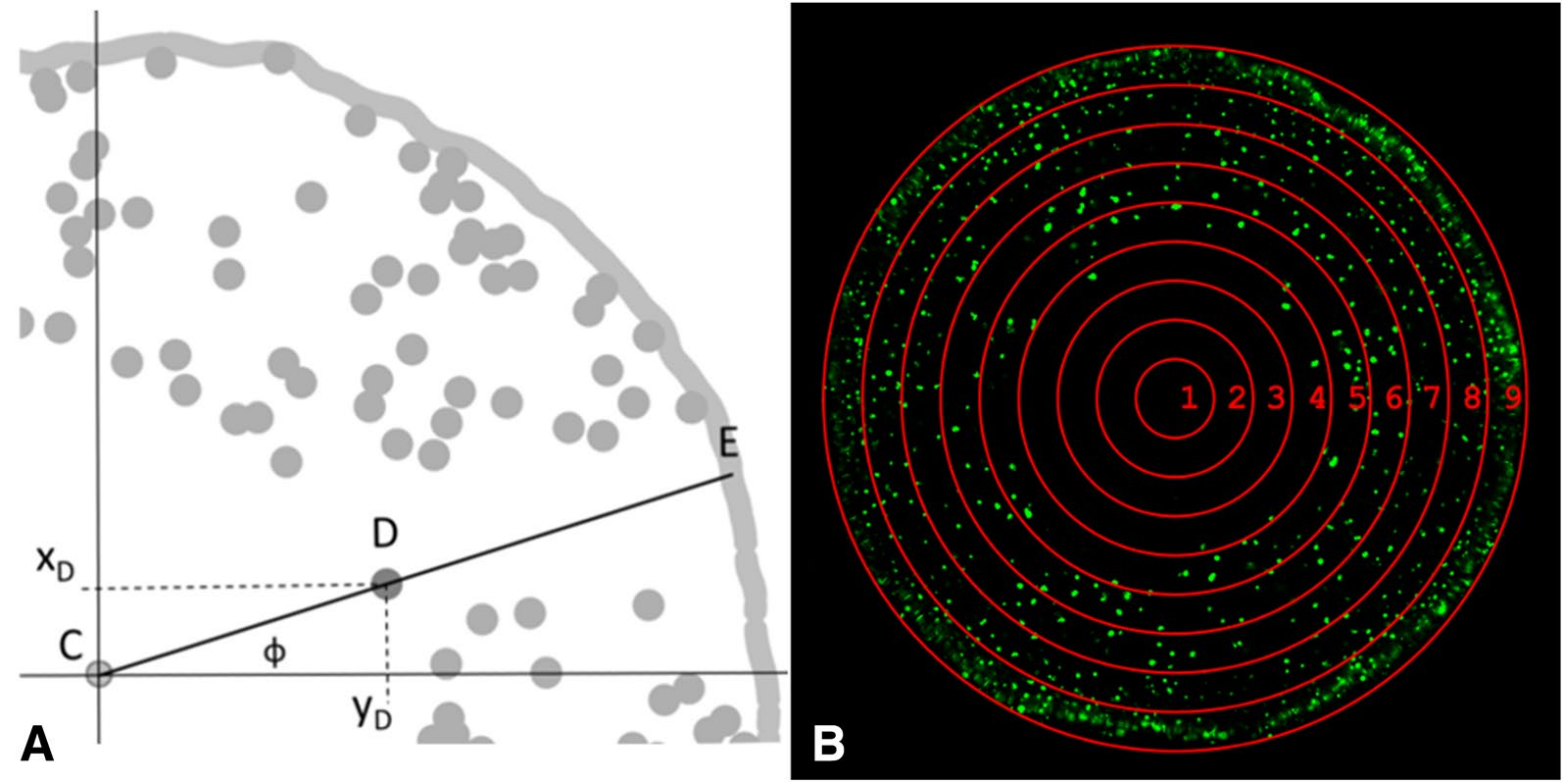
**A** Schematic illustration of a root-slice segment with the identified elements center (C), oil droplets (D, labeled is one oil droplet) and the root edge (E). ϕ indicates the polar angle with C as pole and the x-axis (horizontal line) as polar axis. **B** Generalized illustration of the nine classes (1–9), to which the oil droplets (pp, Formula IV) were assigned based on their relative distance to the center

For data evaluation, the determined x–y-coordinates were adapted and related to each other. The coordinates of the centers were used as new coordinate origins (formula Ia and Ib) and the distances of the oil droplets and the edges related to the center were calculated with Formula II. This was only achievable when the center, the oil droplet and the corresponding point of the root edge all lay on an imaginary line. An example is shown in Fig. 3A. The corresponding point of the root edge was calculated with help of the polar angle, which must be the same for the distance between the center and the oil droplet (CD in Fig. 3A) and for the distance between the center and the corresponding point of the edge (CE in Fig. 3A). The polar angle of both distances was calculated with Formula III. At last, the relative distances of the oil droplets and the root edges to the center were determined (Formula IV). This data was used for the evaluation.

The relative distance data was assigned to one of nine classes with a class width of 11.11%; this led to the interval limits for class 1 = [0–11.10%); class 2 = [11.11–22.21%); class 3 = [22.22–33.32%); class 4 = [33.33–44.43%); class 5 = [44.44–55.54%); class 6 = [55.55–66.65%); class 7 = [66.66–77.76%); class 8 = [77.77–88.87%); class 9 = [88.88–100%]. Figure 3B shows a generalized illustration of the nine classes. Class 1 was always within the central cylinder; class 2 delineated mostly the border of the central cylinder. The area of both classes together was addressed as central cylinder. The classes 3–8 comprised the parenchyma, class 9 the outer cell layers. The area of the classes increased from the center to the edge. Therefore, both the number of oil droplets in each class and the oil droplet density were determined to compare the classes. The density was calculated as the quotient of number of oil droplets over the class area.

For statistical analysis, in each class, the mean number of oil droplets of the root-slices was determined. The distribution of these oil droplets as affected by the horizons, the root diameter fractions, the root classification and the genotypes, were compared with the Friedman-Test and Wilcox-Test. To compare the different factor levels, such as different genotypes (CE), root diameter fractions (RF) or horizons (HZ), the oil droplet density for each root-slice was calculated. An analysis of variance (ANOVA) and *t* Test was performed. For all statistical analyses, significance was given at *p* <  0.05.

Unless otherwise described, the following data represent the mean values over the other factors and their factor levels.

Formula:

Ia $${\text{x}}_{{{\text{mod }}}} = {\text{ x}}_{{\text{i}}} { - }{\text{x}}_{{\text{C}}}$$

Ib $${\text{y}}_{{{\text{mod}}}} = {\text{ y}}_{{\text{i}}} { - }{\text{y}}_{{\text{C}}}$$

II $${\text{r}} = \sqrt {{\text{x}}_{{\bmod }}^{2} + {\text{ y}}_{{\bmod }}^{2} }$$

III $$\upvarphi = \arccos \left( {\frac{{{\text{x}}_{\bmod } }}{\text{r}}} \right)$$

IV $$pp = \frac{{\left( {r_{CD} *100} \right)}}{{r_{CE} }}$$

x_mod,_ y_mod_ = according to the new origin coordinate modified x- and y-coordinate, x_i,_ y_i_ = x- and y-coordinates of oil droplets and root edges, respectively, x_C,_ y_C_ = x- and y-coordinates of centers, r = distance (radius) in pixel points, $\upvarphi$ = polar angle, pp = relative distance, r_CD_ = distance (radius) from center to oil droplet, r_CE_ = distance (radius) from center to root edge

## Results

### Classic light-microscopic imaging

**Fig. 4:**
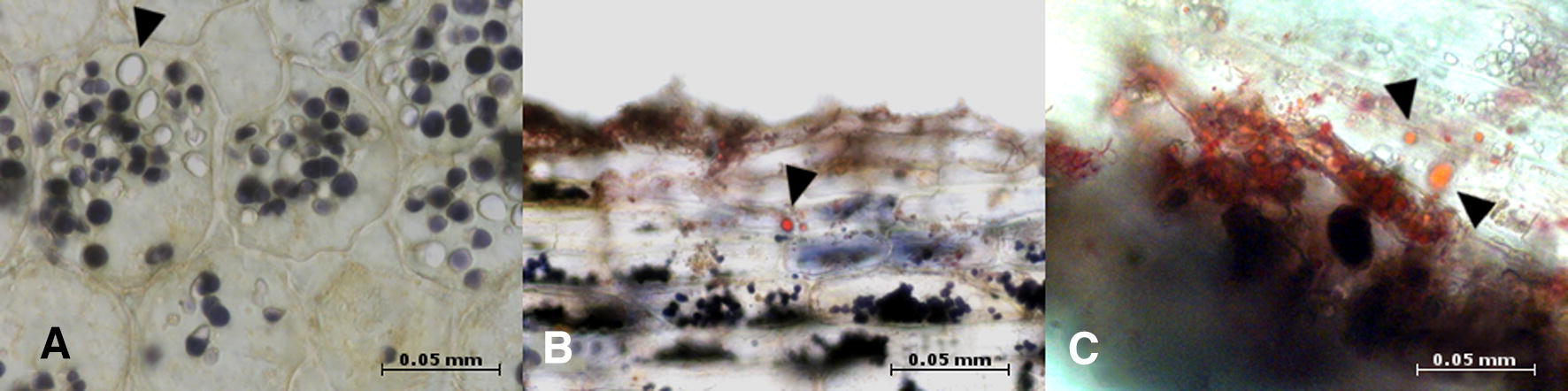
Microscopic images of thin-section slices from fixed and embedded (**A**), and fresh (**B** + **C**) valerian roots. **A** Cross-section through the root parenchyma with stained starch (Lugol’s solution) and intermediary colorless round bodies (black arrow pointer). **B** + **C** Longitudinal-section of the root parenchyma and exodermis. In addition to the colored starch, the red-colored bodies, which were stained by use of a sudan-III-solution, are visible (black arrow pointer)

Figure 4A shows the colored cross-sections of the root parenchyma of a randomly chosen valerian root. Between the colored grains of starch, colorless round bodies are visible. It is assumed that these colorless round bodies contained essential oil (Fig. 4A).

In thin cross-sections and longitudinal-section slices of fresh roots, only few colorless round bodies were stained red by the application of the sudan-III-solution (Fig. 4B, C).

### Fourier-transform infrared (FTIR) imaging

**Fig. 5:**
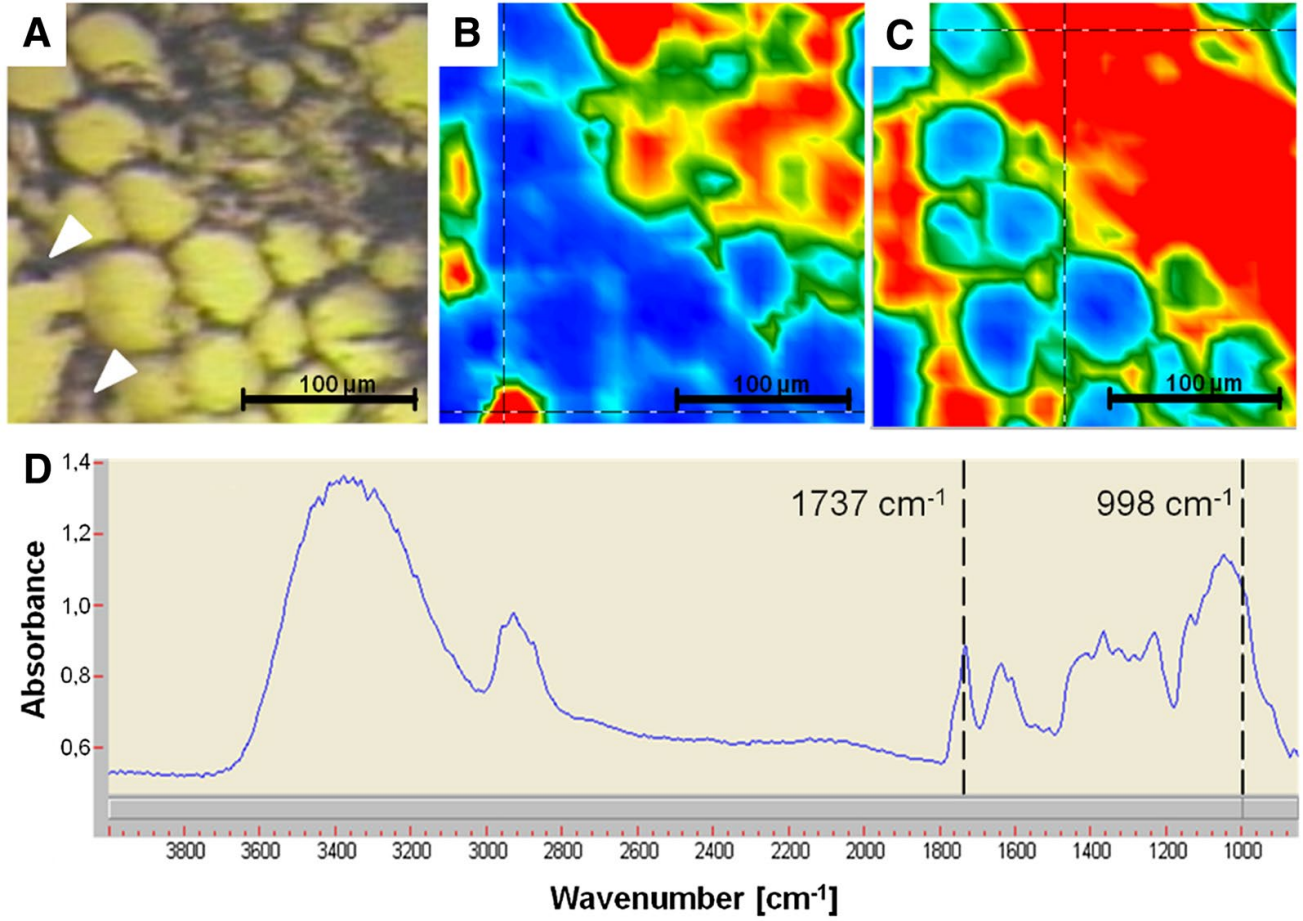
Light-microscopic (**A**) and FTIR images, of valerian root cross section showing high absorbance for mainly bornyl acetate at 1737 cm^−1^ (**B**) and cellulose at 998 cm^−1^ (**C**). The spectrum was taken from the cross mark in the appropriate FTIR images for cellulose (**D**). Colors blue, green, yellow, and red represent increasing content—the warmer the color, the higher the spectral intensity

Figure 5 presents the light-microscopic picture of a thin valerian root slice and the corresponding FTIR images obtained by integration of the absorbance at 1737 cm^−1^ and 998 cm^−1^ (top, from left to right). Whereas the signal at 1737 cm^−1^ can be tentatively assigned to bornyl acetate, the absorption at 998 cm^−1^ mainly represents cellulose matrix. The spectrum presented in Fig. 5D was taken at the crossed lines shown in the integration map for 998 cm^−1^ (Fig. 5C). As indicated by arrows in the light microscopic picture, two intact oil bodies might be identified due to the intensive absorption at 1737 cm^−1^. The red colored part in the right upper corner of that image might be the result of destroyed oil bodies and smeared essential oil due to microtome preparation of the root slide. Generally, in the sections used for FTIR imaging, it was very rare to find intact colorless bodies for which high absorbance around 1737 cm^−1^ was observed. Unfortunately, the presence of oil bodies could not be confirmed by adjacent staining experiments.

### Fluorescence-microscopic imaging and mapping of different root material

A total of 678 root-slices of valerian were evaluated. The number of root-slices was distributed quite evenly among the different levels of the factors: cloned elites (CE), root diameter and classification, root horizon (HZ). However, fewer root-slices were analyzed in regard to the root diameter fraction RF4, because the thin-rooted cloned elites did not form roots with a diameter greater than 4 mm (Tables 1, 2).

**Table 2:** Number of slices, sum of oil droplets and mean number of oil droplets in the root-slices of four cloned elites (CE1, CE2, CE3, CE4), of two root classifications (thin- and thick-rooted), of four root diameter fractions (RF1, RF2, RF3, RF4) and of three horizons (HZ1,HZ2, HZ3)

	Number of root-slices	Sum of oil droplets	Mean number ± standard deviation of oil droplets per root-slice
*Cloned elites*			
CE1	176	34,665	197 ± 84
CE2	162	43,466	268 ± 149
CE3	171	61,419	359 ± 255
CE4	178	53,666	301 ± 168
*Root classification*			
Thin	338	78,131	231 ± 125
Thick	349	115,085	330 ± 217
*Root diameter fractions*			
RF1	211	37,422	177 ± 87
RF2	195	42,647	219 ± 98
RF3	210	77,602	370 ± 192
RF4	71^a^	35,545	501 ± 239
*Horizons*			
HZ1	206	68,904	334 ± 225
HZ2	246	74,994	305 ± 184
HZ3	235	49,318	210 ± 107

^a^The root diameter fraction was only formed by CE3 and CE4 (Table 1)

**Fig. 6:**
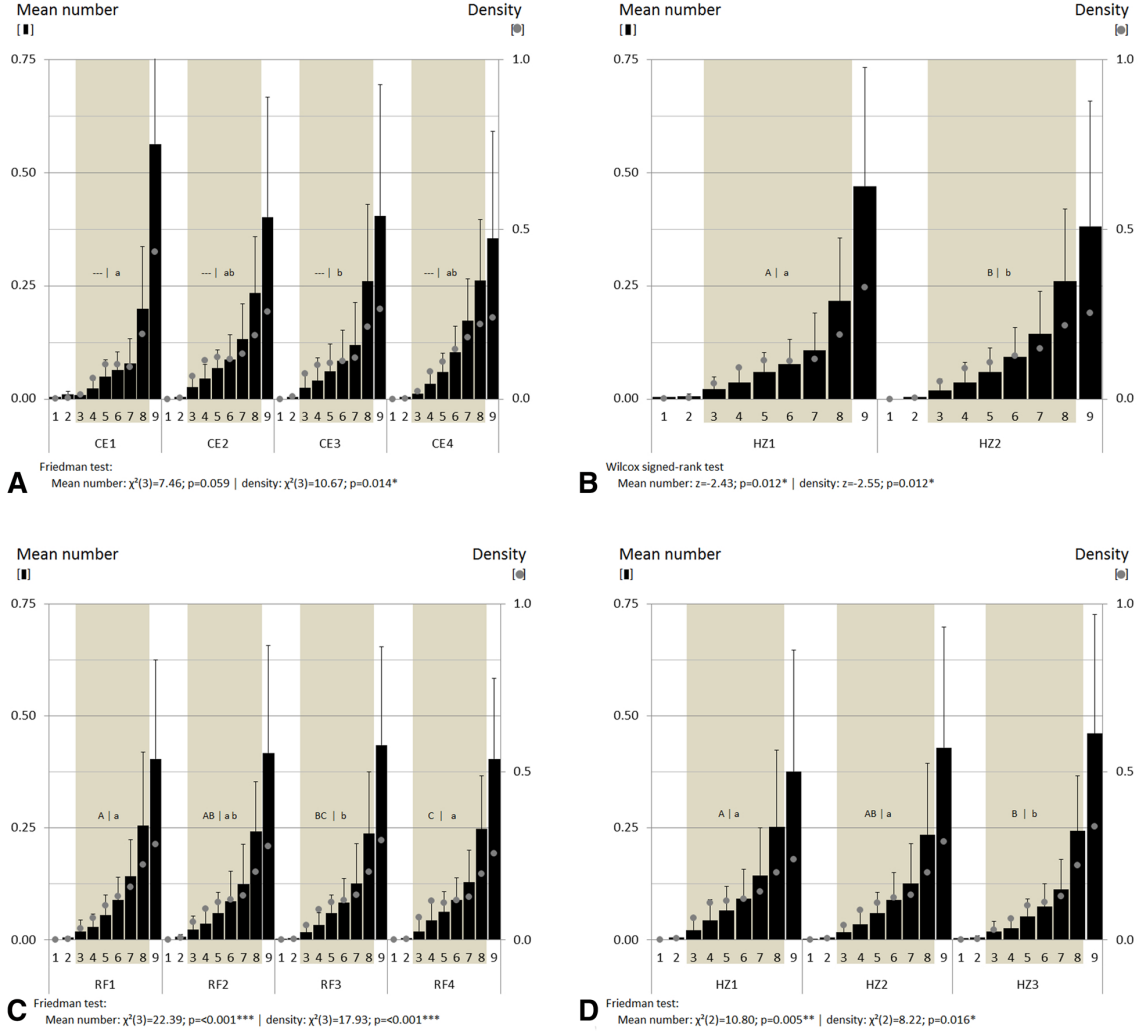
Mean number (columns) and density (points; mean number/class area) of oil droplets, determined from root thin section slices of valerian and allocated in nine classes (1–9). Shown are four cloned elites (CE1, CE2, CE3, CE4) (**A**), two root classifications (thin- and thick-rooted) (**B**), four root diameter fractions (RF1, RF2, RF3, RF4) (**C**) and three root horizons (HZ1, HZ2, HZ3) (**D**). Vertical lines: standard deviation. Significant codes: *< 0.05, **< 0.01, ***< 0.001. The same capital and small form letters mark that the oil droplet mean numbers and the densities are equal (*p* <  0.05, SNK), respectively. The colored background marks the classes of the parenchyma. Further details of the cloned elites, the root classification, the root diameter fractions, the horizons and the parenchyma are shown in the text

The distribution of the mean number of oil droplets in each factor level was approximately constant (Fig. 6). Significant differences between the distributions of the factor levels of root classification (Wilcox: *p* = 0.012), root diameter fraction and horizon (Friedman: *p* < 0.001 and *p* = 0.005, resp.) were especially visible in class nine (Fig. 6B, C). In general, the mean number of oil droplets increased from the center to the outer cell layers. The central cylinder was almost free of oil droplets, whereas, the parenchyma included 57% (42–64%) of the mean number of oil droplets on average. In the outer cell layer, completely covered by class nine, 43% (36–56%) of the mean number of oil droplets were present, on average.

**Fig. 7:**
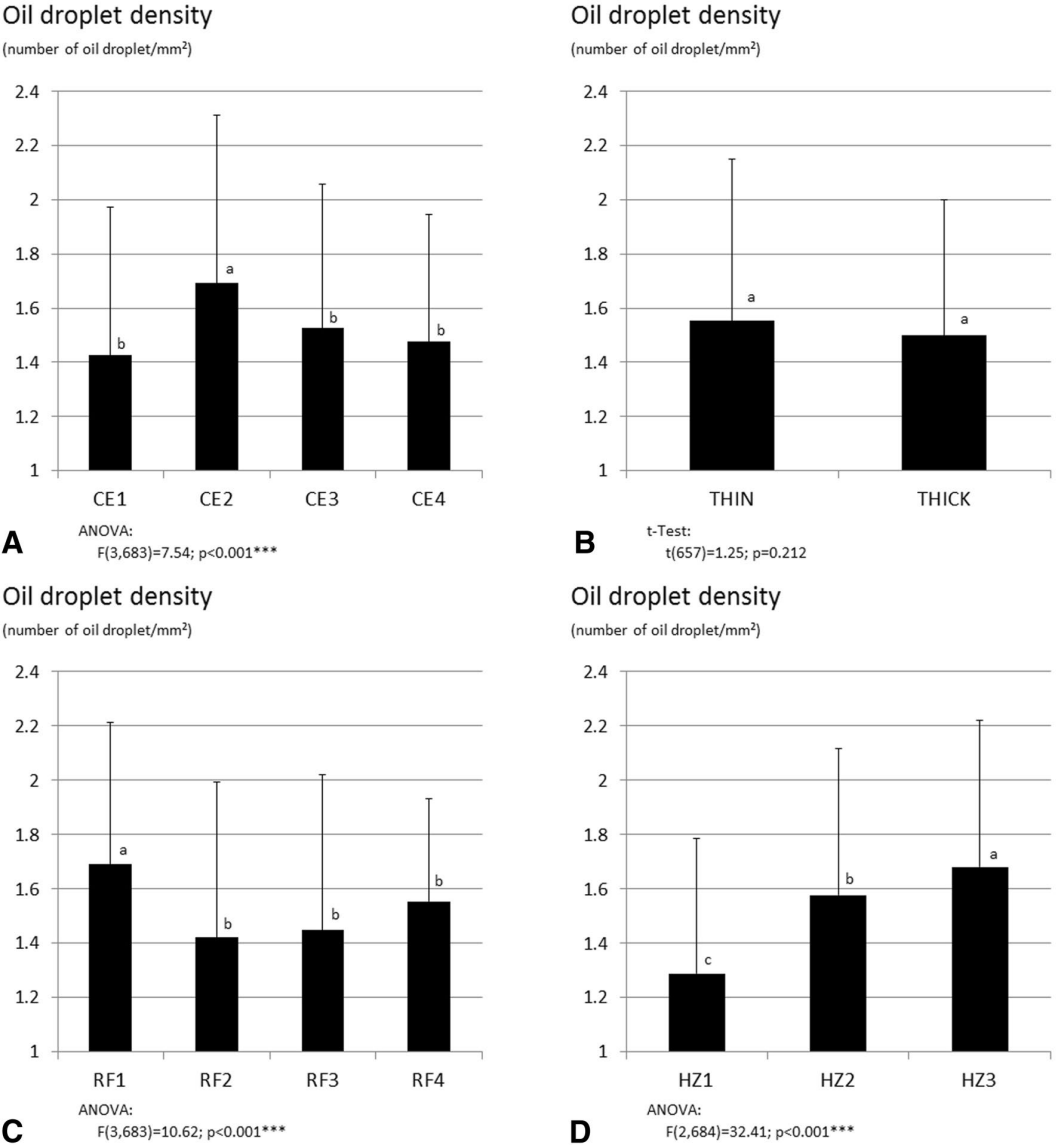
Mean oil droplet density (number of oil droplets/mm^2^), determined from root thin section slices of valerian. Shown are four cloned elites (CE1, CE2, CE3, CE4) (**A**), two root classifications (thin- and thick-rooted) (**B**), four root diameter fractions (RF1, RF2, RF3, RF4) (**C**) and three horizons (HZ1, HZ2, HZ3) (**D**). Further details of the cloned elites, the root classification, the root diameter fractions and horizons are shown in the text. Vertical lines: standard deviation. Significant codes: *< 0.05, **< 0.01, ***< 0.001. Different small form letters mark significant differences between the oil droplet densities (*p* <  0.05, SNK). Number of root slices given in Table 2

To consider the varying areas of the classes, the oil droplet density was a more suitable parameter to compare the classes than the number of oil droplets. A constant density was not found. Similar to the mean numbers, the density of oil droplets also rose up from class one to class nine (Fig. 7). Each factor showed a significant effect on the density of oil droplets (Friedman: CE *p* = 0.014; RF *p* < 0.001; HZ *p* = 0.016; Wilcox: root classification *p* = 0.012).

**Fig. 8:**
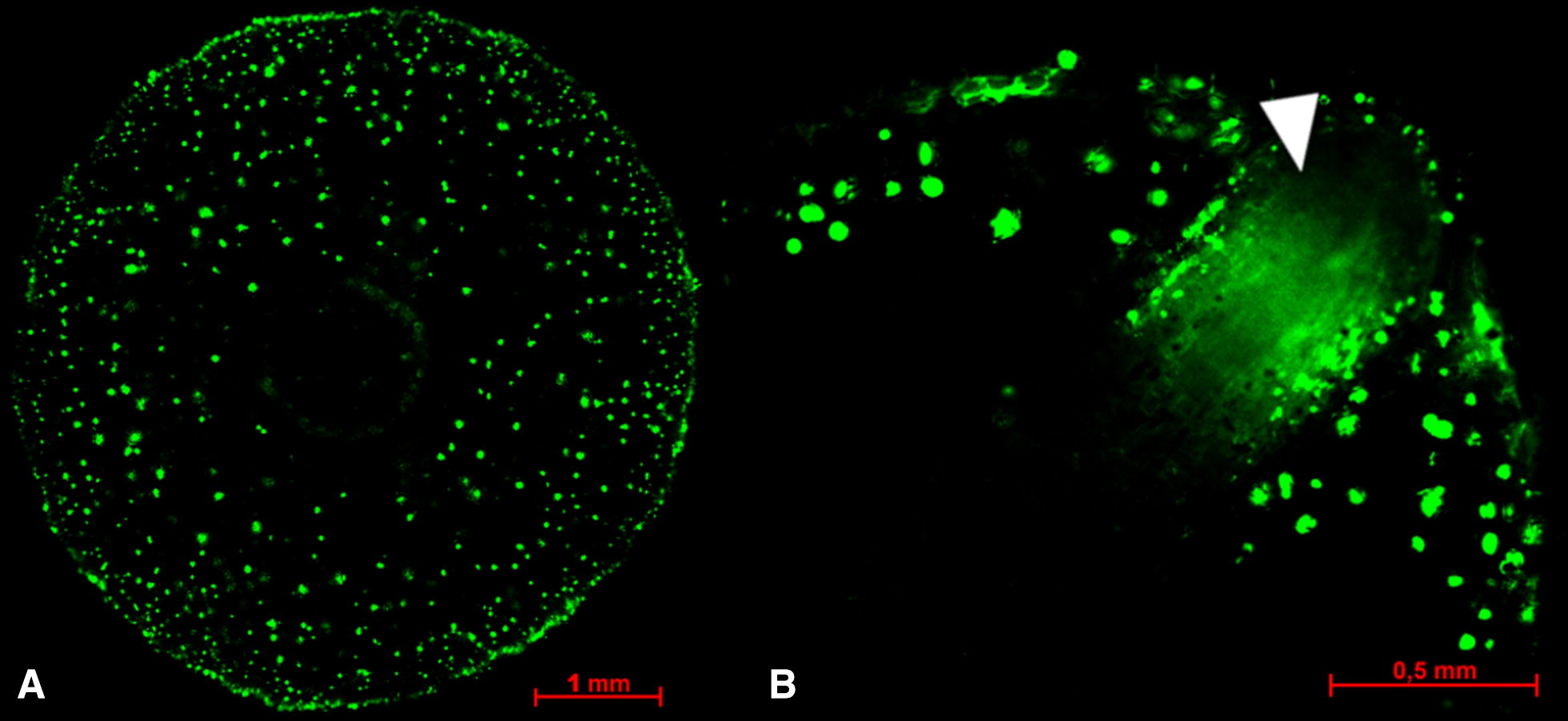
Fluorescent oil droplets in cross-sections of valerian roots: adventitious root (**A**), growing lateral root breaking through the exodermis (**B**, white arrow pointer)

The root diameter of each factor level affected the number of oil droplets. Thicker root-slices contained more oil droplets, as presented in Table 2. This meant, ultimately, that the number of oil droplets increased with root thickness, root diameter and in the upper located root horizons as compared to the lower horizon. As shown for the class comparison, the density of oil droplets allowed for a better comparison between factor levels. Among the cloned elites, CE2 showed the highest oil droplet density (ANOVA *p* <  0.001; Fig. 8C). RF1 was the root diameter fraction with the highest oil droplet density (ANOVA *p* < 0.001; Fig. 8C), whereas each horizon showed a significantly different oil droplet density (ANOVA *p* <  0.001; Fig. 8D), with an increasing oil droplet density from HZ1 to HZ3.

Figure 8A shows fluorescent oil droplets in a representative root cross-section. Based on the image area of the oil droplets, the size of oil droplets allocated to classes 3–7 was approximately 72% larger than the size of the oil droplets allocated to classes 1 and 2. This was more or less recognizable for all root-slices, but was not examined in detail. In the outer cell layer of young lateral roots we could already find oil droplets (Fig. 8B).

## Discussion

### Histochemical structures and methods (k, kk)

#### Comparable histological structures were found as reported for valerian in literature

We compared our breeding plant material with the existing literature information by using classic light-microscopic imaging techniques. Many grains of starch are densely arranged in the cells and distributed over the entire parenchyma [20–22, 39]. In longitudinal-section slices from fresh roots, oil droplets were visible, who are located near the cell-wall [20, 40]. Large-lumen cells of the exodermis were observed, however, the filling of the cell with one oil droplet [20, 21, 39] could not be detected.

The staining of the round bodies with sudan-III-solution worked well in the fresh roots, but not in the embedded roots. Probably, the essential oil volatilized due to either the use of ethanol during the embedding process or due to the ethanol-containing sudan-III-solution itself. Similar effects were observed by Fridvalszky [21] and Violon et al. [22], who concluded hereupon that the lipid bodies contained ‘volatile oil’.

#### The colorless round bodies are filled with essential oil

Generally, FTIR imaging can provide information about the distribution of different plant constituents without destructing the plant constituent containing cell structure. These constituents include lipids, carbohydrates, and lignin, as well as secondary metabolites, e.g. terpenoids [41].

Nevertheless, the local accumulation of the strong absorbance at 1737 cm^−1^ tentatively assignable to bornyl acetate indicates an essential oil distribution in distinct cellular structures. As bornyl acetate is a principal component of the essential oil of valerian, the results from FTIR imaging confirms the theory that essential oil is located within the colorless bodies. However, only very few intact oil droplets were found in the thin cryo-sections. The preparation of thin root slides of a thickness below 0.01 mm usually resulted in disruption of the oil bodies and smearing of the essential oil. Therefore, the results of FTIR imaging have to be seen as basic studies combining visual images of the sample with chemical information. The authors’ own previous studies on valerian root sections performed with FT-Raman spectroscopy did not deliver additional information about essential oil distribution. Only the differentiation of various root tissue mainly based on carbohydrate profile was obtained and with the instrument used, the local resolution was limited to around 150 µm (data not shown).

Modern Raman microscopes achieve a local resolution below 1 µm depending on the laser and aperture used. To gather suitable signal intensity, the Raman laser needs to be exactly focused, which demands for the extremely thin root slices. With application of the necessary laser power, the brownish root material often started burning and the spectra showed high fluorescence appearance. A reduction in laser power resulted in a lack of signals. Therefore, if the analytes don’t contain Raman active bonds (e.g. C–C double bonds in carotenoids) and the sample is colored (as the majority of plant derived material is), Raman spectroscopy investigations are technically challenging.

In recent years, another vibrational spectroscopy method gained attention in plant analysis. Hyperspectral (near infrared) spectroscopy imaging can be used for various analytical purposes as species identification, disease detection or nutrient quantification, but is focused on macroscopic samples due to the relatively coarse spatial resolution of several 100 µm [42–45].

Taking into account the above described characteristics of each method, FTIR imaging in combination with subsequent staining to affirm the preliminary results, seems to be the most favorable analytical strategy. The resulting FTIR distribution maps showing the strong C = O vibration at 1737 cm^−1^ confirm the results of staining experiments and fluorescence-microscopy.

Due to the failed repeatability of the FTIR imaging of oil droplets, the more robust method of fluorescence-microscopy was chosen to visualize the oil droplets.

#### Relative distances and class width are suitable to compare the root diameter fractions and root horizons

The oil droplet position was calculated as relative distance from the root center to the root edge. The relative distances were allocated to nine width classes, which allowed a comparison between the root diameter fractions and root depth (horizons), regardless of the real root diameter of the considered root slice. The root slices seldom showed a circular shape. Often, the root slices were oval or had coves. An absolute and constant class width would not have been appropriate, because the border of class nine would not necessarily be congruent with the edge of the root slice. Further information concerning the oil droplet distribution would be achieved if densities of the classes are calculated based on absolute diameters and cross-section areas. For our purposes, the absolute oil droplet densities of the total cross-section areas were sufficient.

### Meaning of oil droplet distribution and density for valerian breeding and production (i, ii)

With the current investigations, we were able to show that oil droplets can be found in the whole parenchyma. These results are in good agreement with previous studies of Violon et al. [22] and Szentpetery et al. [40]. Besides the identification of the oil droplets, the number and density of oil droplets are also now available for different varieties, different root diameters on the same plant or for different positions along the roots.

#### Conclusions about the relationship of oil droplet occurrence and essential oil content of genotypes are limited

Chemical-analytical data does not exist for the currently studied plant tissue, but genetically identical plant material has been investigated analytically by Penzkofer et al. [19]. We tried to compare and re-estimate the results of both studies.

The comparison of the four clones gives only limited information for the relationship between oil droplet occurrence and oil content, due to the low number of clones, the limited genetic variability among clones and the environmental and year’s effect, which all potentially influence the essential oil content [46, 47]. Even Penzkofer et al. [19] could only detect trends in this regard, but identified clone CE2 as the one with the highest essential oil content. This did not coincide with our essential oil mapping data. Concerning the number of oil droplets per root cross-section, clone CE3 shows the highest values, followed by clone CE4 and CE2 (Table 2). Thus, the number of oil droplets was not a suitable identifier for the essential oil content. This may be due to the varying oil droplet sizes in the inner parenchyma.

In contrast, the essential oil droplet density based on total root cross-section area is more informative. Clone CE2 had the highest oil droplet number/mm^2^ (Fig. 7) and it showed the highest oil content in the study by Penzkofer et al. [19]. However, to evaluate the relationship between essential oil droplet density and essential oil content, more data is needed.

#### The number and density of oil droplets are not negatively related to the root thickness

The mapping results showed an expected increase of the number of oil droplets per cross-section from thinner to thicker roots (Table 2). For the fractions with low to high root diameters (RF1-RF3), the number of oil droplets was in accordance with the essential oil content results of Penzkofer et al. [19]. The fraction with the largest root diameter (RF4) showed the highest number of oil droplets in our study, but the lowest essential oil content in their study. It has to be noted that the essential oil content was determined by a very small number of data points and comprised only the clone CE4, because the other clones did not develop roots of this diameter. Considering CE4 only, the oil droplet number was the same for thick and very thick roots (RF3 and RF4, respectively).

The oil droplet density of total root cross-sections was highest in the thin roots (RF1) and lower in medium to thick roots (RF2-4). Thus, no linear relationship between oil drop density and root thickness can be derived. The high oil droplet density in thin roots may partially be an effect of root age, assuming that a higher portion of young roots occurred in this root fraction, and that oil droplets are formed at a very early stage of development, a phenomenon we were able to see with our image of a young lateral root. However, root age may not entirely explain the difference in oil droplet density between RF1 and RF2 as there was no difference between RF2 and RF3.

Concerning the breeding target of thicker roots, we found a positive relationship between root thickness and number of oil droplets per root cross-section. We did not find a negative relationship between root thickness and oil droplet density, especially when medium to very thick roots were considered. Consequently, there is a potential for selection of thick roots that have high oil droplet densities and, therefore, also likely high essential oil content. This is supported by the experience that many inbred lines with coarse roots and high essential oil content could be derived from clone CE4.

#### Oil droplet distribution should be considered in cultivation, harvesting and breeding

Considering that 43% of the oil droplets were localized in the outer section of the roots (class nine), it is evident that a careful root harvest and processing, without damaging the root surface, is important. This also means that a high portion of oil droplets are located in the inner parenchyma, information which is important for valerian cultivation, as well as for breeding selection.

Heindl and Hoppe [48] reported that intense and long washing of valerian roots leads to a loss of secondary compounds. Therefore, the more essential oil is stored in the inner parenchyma, the lower should be the losses during the processing, and the higher is the processing stability of the roots.

One of the main breeding targets in the past was increasing the essential oil content [47]. With this target in mind, the losses of oil during processing and storage are minor. We now show that in different single plants, represented by the clones of elites, different essential oil distributions exist. Choosing plant types with steadily increasing oil droplet density curves instead of plants with exponentially increasing oil droplet densities towards the root edge implies that a higher portion of the oil will be located in the inner parenchyma. Moreover, processing stability and possibly, also, storage stability of valerian roots can be increased. Our imaging and mapping method, in combination with the calculated density curves, can serve as a selection tool for identifying suitable plants.

The comparison of horizons provides further information about the reliability of the production process. The horizons represent harvest depths: The greater the depth, the thinner the formed roots, and the greater the likelihood that they will be more easily lost during the harvesting process.

These horizons contain a lower number of oil droplets. Thus, the harvest of the root parts in deeper soil layers does not have to be exhaustive in order to obtain high essential oil contents in the root drug, especially when 96% of the root mass is located in the topmost 10 cm of the ground [49].

## Conclusion

For the first time, the essential oil distribution in entire valerian root cross-sections of different varieties, root thicknesses and root horizons were visualized, applying an imaging and mapping fluorescence-microscopy technique. The applied methods of FTIR spectroscopy and fluorescence-microscopy allowed for the determination of oil droplets as clearly differentiated structures.

Our results give insight into the cross-sectional essential oil distribution at valerian harvest time. Although the existing natural variability and the limited plant material investigated in this study only allow for a brief overview, some aspects derived from the results could be used for further investigations and for future breeding work:

1. The number, density and distribution of essential oil droplets of genetically different plant material vary and allow for the selection of suitable plant material for breeding purposes, independent of root thickness.
2. The breeding plant material should generally exhibit a high oil droplet density level as this is one of the factors for the essential oil content.
3. A high and homogeneous oil droplet density should be aspired in the inner parenchyma in order to avoid essential oil losses during harvesting and post-harvest processes.

It still remains unclear which oil droplet characteristic is preferred as a selection trait. In consequence, a compromise between the absolute oil droplet density and the oil droplet density curve must be found, while at the same time, considering root thickness and individual genetic background.

Finally, a careful, but not necessarily exhaustive harvest, as well as careful post-harvest procedures, confirmed as best methods for conserving the essential oil and obtaining a high quality of the valerian root drug.

## Additional file

**Additional file 1.** Analyzation steps and software macros for localization of the oil droplets, root-slice area and edges, as well as the root-slice center.

## Abbreviations

CE: cloned elite plant
RF: root thickness fraction
HZ: root horizon
FTIR: Fourier-transform infrared
