# Supplementary material for: Characterization of essential oil distribution in the root cross-section of Valeriana officinalis L. s.l. by using histological imaging techniques
Source: Plant Methods. 2018 Jun 4;14:41. doi: 10.1186/s13007-018-0309-4 (PMC5985564; doi:10.1186/s13007-018-0309-4)
Supplement: Supplementary file 1 — Additional file 1. Analyzation steps and software macros for localization of the oil droplets, root-slice area and edges, as well as the root-slice center. [file 13007_2018_309_MOESM1_ESM.pdf]

Characterization of essential oil distribution in the root cross-section of *Valeriana officinalis* L. s.l. by using histological imaging technique

Penzkofer M, Baron A, Naumann A, Krähmer A, Schulz H, Heuberger H

Analyzation steps and software macros for localization of the
① oil droplets, ② root-slice area and edges, as well as ③the root-slice center.

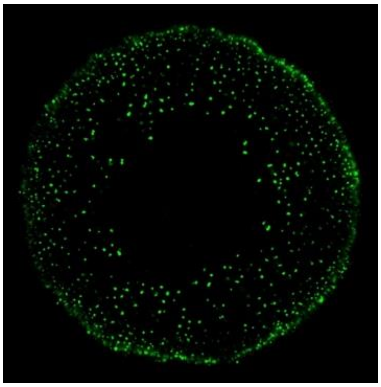

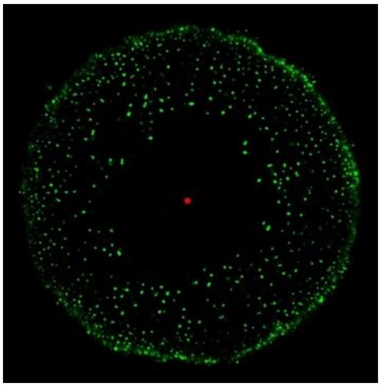

①

```
//*** Macro Oil Droplets ***
run("8-bit");
setThreshold(80, 255);
setOption("BlackBackground",
  false);
run("Make Binary", "thresholded remaining
black");
run("Dilate");
run("Median...", "radius=4");
run("Analyze Particles...",
   "size=350-7000 circularity=0.50-
   1.00 show=Masks display clear");
```

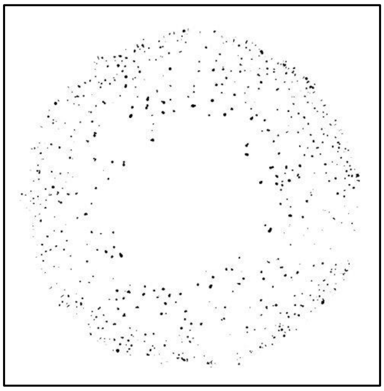

②

```
//***  Macro Area and Edge ***
run("8-bit");
setAutoThreshold("Huang");
setThreshold(0, 0);
run("Make Binary");
run("Invert");
run("Gaussian Blur...", "sigma=20");
setAutoThreshold("Huang");
setThreshold(1, 255);
run("Make Binary");
run("Fill Holes");
run("Create Selection");
run("Enlarge...", "enlarge=-100");
run("Enlarge...", "enlarge=100");
setBackgroundColor(255, 255, 255);
run("Clear Outside");
run("Select None");
run("Minimum...", "radius=60");
run("Create Selection");
run("Set Measurements...", "area
   centroid center perimeter feret's display
redirect=None decimal=3");
run("Analyze Particles...",
   "size=100000-Infinity
   show=Outlines display" );
```

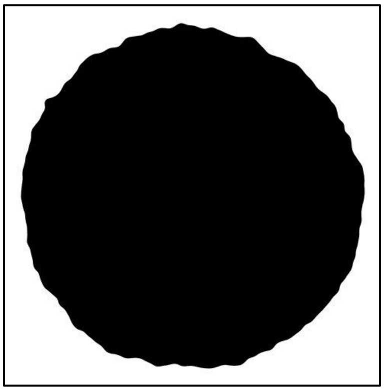

③

```
//*** Macro Center ***
run("Split Channels");
selectWindow(img_rgb_title + "
  (red)");
setThreshold(20, 255);
run("Make Binary", "thresholded remaining
black");
run("Analyze Particles...",
  "size=500-Infinity circularity=0.80
  -1.00 show=Masks display clear");
```

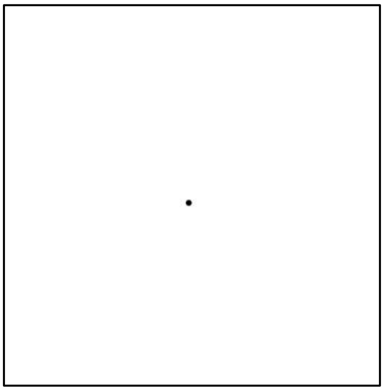

Software: ImageJ, Language: IJ1Macro
